# Association between the atherogenic index of plasma and the non-high-density lipoprotein cholesterol to high-density lipoprotein cholesterol ratio with early neurological deterioration after thrombolysis

**DOI:** 10.3389/fneur.2025.1619727

**Published:** 2025-08-28

**Authors:** Yan Jiang, Mingzhu Deng, Kangping Song, Zhen Wang, Wei Xu, Tieqiao Feng, Sufen Chen, Can Wan, Xiugui Ma, Fangyi Li

**Affiliations:** ^1^Department of Neurology, The Second People’s Hospital of Hunan Province (Brain Hospital of Hunan Province), Changsha, China; ^2^Department of Neurology, The Affiliated Changsha Central Hospital, Hengyang Medical School, University of South China, Changsha, China

**Keywords:** acute ischemic stroke, atherogenic index of plasma, non-high-density lipoprotein cholesterol to high-density lipoprotein cholesterol ratio, early neurological deterioration, lipid metabolism, risk factor

## Abstract

**Background:**

The atherogenic index of plasma (AIP) and non-high-density lipoprotein cholesterol to high-density lipoprotein cholesterol ratio (NHHR) are newly developed markers of lipid and glucose metabolism. Nevertheless, the associations between the AIP or NHHR and early neurological deterioration (END) following thrombolysis in patients with acute ischemic stroke (AIS) remain ambiguous.

**Methods:**

1,323 AIS patients who had intravenous thrombolysis between January 2018 and October 2024 were retrospectively analyzed. An increase in the National Institutes of Health Stroke Scale (NIHSS) score of > 4 within 24 h following thrombolysis was considered post-thrombolysis END. Logistic regression analysis was conducted to investigate the associations between the AIP and NHHR with post-thrombolysis END. Receiver operating characteristic (ROC) analysis was employed to evaluate the AIP and NHHR capacity to differentiate post-thrombolysis END.

**Results:**

Of the 1,323 patients who were recruited, 1,125 (85.03%) had non-END and 198 (14.97%) had post-thrombolysis END. A binary logistic regression model demonstrated that the AIP [odds ratio (OR), 1.657; 95% confidence interval (CI) 1.441–1.875, *p* < 0.001] and NHHR (OR, 1.519; 95% CI, 1.342–1.812, *p* < 0.001) were independent factors for post-thrombolysis END. The area under the curve (AUC) values for the AIP, NHHR, and AIP combined with the NHHR for post-thrombolysis END were 0.753, 0.678, and 0.795, respectively.

**Conclusion:**

Our study suggests that the AIP and NHHR could be used as prognostic indicators to predict post-thrombolysis END.

## Introduction

Stroke is a significant global public health challenge, characterized by high morbidity and mortality rates (1), with acute ischemic stroke (AIS) constituting approximately 70–80% of all strokes (2). Intravenous recombinant tissue plasminogen activator in the early phase (≤4.5 h) has been proven as the first-line treatment for AIS (3, 4). Some patients may have favorable long-term outcomes after vascular thrombolysis and recanalization, but others will still experience early neurological deterioration (END), which is when their neurological deficits and symptoms get worse within 24 h of thrombolysis (5, 6). For AIS patients, END is associated with poor long-term outcomes and an increased risk of death and disability (7, 8). To enhance the clinical prognosis of patients, it is crucial to identify predictors of post-thrombolysis END, identify high-risk stroke patients early, and assist in assessing future preventative initiatives.

Given the role of lipid metabolism in vascular pathophysiology and inflammation, lipid-derived indices such as atherogenic index of plasma (AIP) and non-high-density lipoprotein cholesterol to high-density lipoprotein cholesterol ratio (NHHR) have gained attention as potential predictors of adverse cerebrovascular outcomes. The AIP and NHHR are emerging developed markers of lipid and glucose metabolism. The AIP is the logarithmic transformation of the ratio of the level of triglyceride (TG) to the level of high-density lipoprotein-cholesterol (HDL-C), that is, log (TG/HDL-C) (9). Previous studies have pointed out the logarithmic conversion of the ratio of TG to HDL-C was negatively correlated with the diameter of low density lipoprotein particles, indirectly reflecting small and dense low density lipoprotein cholesterol (sdLDLC), that is highly sensitive to underlying insulin resistance, making it an excellent indirect biomarker for glucose metabolism dysregulation risk (10, 11). NHHR is an emerging comprehensive indicator of atherosclerotic lipid. Non-high-density lipoprotein cholesterol is considered a pivotal contributor to cardiovascular disease (12). HDL-C exerting protective effects primarily through cholesterol transfer reversal, anti-inflammatory, antioxidant, anti-apoptotic, and vasodilatory mechanisms (13). AIP has been employed to evaluate metabolic syndrome, insulin resistance, and atherogenic dyslipidemia (14–16). The early identification of individuals with a high risk of cardiovascular disease due to aberrant glucose metabolism will be facilitated by the monitoring of long-term AIP change (17). Multiple epidemiological studies have indicated a significant association between AIP and coronary heart disease (18) as well as symptomatic carotid stenosis (19). A high cumulative AIP is associated with an increased risk of ischemic stroke (20). AIS patients were prospectively enrolled, revealing that elevated AIP correlated with unfavorable outcomes in all stroke patients (21). A recent study also found that a higher AIP index was linked to END in AIS patients who had mechanical thrombectomy treatment for an urgent large vessel occlusion (22). However, there is no data explicitly evaluating the relationship between AIP and END following thrombolysis in individuals with AIS. NHHR is a novel, comprehensive biomarker of atherosclerotic lipids that possesses significant predictive value in individuals with cardiovascular disease (23–26). Despite the increasingly significant data that emphasizes the significance of the NHHR, its connection to stroke is remains insufficiently characterized. A prior study indicated that NHHR is associated with an increased prevalence of stroke and may become a new predictor of stroke among adults in the USA (27). However, to date, no study has simultaneously examined the association of AIP and NHHR with post-thrombolysis END following intravenous thrombolysis in AIS patients, particularly within the Asian population.

END is a clinically severe complication, leading to poor prognosis and causing a heavy burden on patients and families (28, 29). Additionally, the long-term prognosis of patients is correlated with the early neurological outcome following thrombolysis (8, 30). Therefore, the present study aimed to investigate the associations of AIP and NHHR with the risk of early neurological deterioration in AIS patients treated with intravenous thrombolysis.

## Materials and methods

### Study design and participants

**Figure 1:**
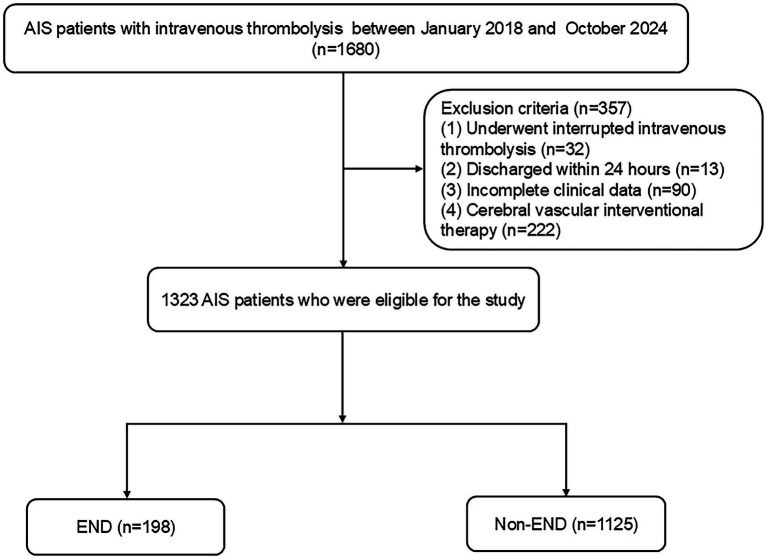
Study flow diagram. AIS, acute ischemic stroke; END, early neurological deterioration.

This multicenter retrospective analysis identified consecutive AIS patients who underwent intravenous thrombolysis within 4.5 h from two urban hospitals in China (center 1: Changsha Central Hospital; center 2: Hunan Province’s Second People’s Hospital). AIS was diagnosed based on the subsequent criteria: (1) Sudden onset; (2) localized neurological impairments (such as weakness or numbness on one side of the face or limb, language difficulties, etc.), with some presenting as generalized neurological deficits; (3) imaging showing lesions or symptoms/signs persisting for over 24 h; (4) elimination of nonvascular etiologies; and (5) brain CT/MRI to rule out cerebral hemorrhage (31). The inclusion criteria included: (1) admission within 4.5 h post-onset; (2) treatment with intravenous thrombolysis using r-tPA; and (3) participants aged 18 years or older. The exclusion criteria for patients included: (1) discharge within 24 h; (2) interruption of intravenous thrombolysis due to severe side effects; (3) incomplete clinical data; and (4) cerebral vascular interventional therapy. The Ethics Committee of Changsha Central Hospital and the Second People’s Hospital of Hunan Province authorized this study. From January 2018 to October 2024, 1,323 AIS patients were enrolled (Figure 1).

### Data collection

The clinical evaluations were conducted by expert neurologists in a blinded manner. This study used secondary data from medical records. We collected data from each participant, which including age, sex, body mass index, stroke risk factors (hypertension, diabetes mellitus, atrial fibrillation, coronary artery disease, current alcohol consumption, and smoking), and clinical features (severity of stroke at admission, admission systolic blood pressure (SBP) and diastolic blood pressure (DBP), onset to treatment time (OTT), and stroke subtypes). The stroke subtype was identified using transcranial Doppler, carotid ultrasonography, echocardiography, electrocardiography, magnetic resonance imaging, and computed tomography. The parameters of the Trial of Organization 10,172 in Acute Stroke Treatment were used to categorize the stroke subtype. The demographic data, baseline clinical parameters, clinical diagnoses, and treatment plans were carefully collected using a standardized case-report form.

Blood samples were collected from all patients at 6–7 a.m. following a minimum fasting period of 8 h. For standard blood tests, two milliliters of EDTA-anticoagulated whole blood were utilized (BZ6800, China). For the standard biochemical analysis, 5 mL of blood containing coagulant were employed (HITACHI 7600, Japan). Triglycerides (TG), total cholesterol (TC), neutrophils, platelets, monocytes, white blood cells (WBC), low-density lipoprotein cholesterol (LDL-C), high-density lipoprotein cholesterol (HDL-C), and fasting blood glucose (FBG) levels were assessed in blood samples. All the indicators were tested using commercial kits, which were operated by qualified professionals in accordance with the specifications. Each of the blood tests was performed three times. The following formula was used to calculate AIP: Log [TG (mmol/L)/HDL-C (mmol/L)] (9). The NHHR index was computed using the following calculation: (TC-HDL-C)/HDL-C (27).

### Definition of post-thrombolysis early neurological deterioration and symptomatic intracranial hemorrhage

The certified neurologists at both centers were blinded to our study and underwent standardized training for the assessment of NIHSS scores. An increase in the National Institutes of Health Stroke Scale (NIHSS) score of >4 within 24 h following thrombolysis was considered post-thrombolysis END (32, 33). Within 24 h following thrombolysis, a clinical deterioration of at least four points on the NIHSS score was considered symptomatic intracranial hemorrhage (sICH), which could be caused by an intraventricular hemorrhage, subarachnoid hemorrhage, or parenchymal hematoma (34).

### Statistical analysis

Data analysis was conducted using SPSS 25.0 (IBM SPSS Statistics software, Version 25.0). The Kolmogorov–Smirnov test was employed to assess if the distribution of all the data was normal. If the continuous variables were regularly distributed, they were shown as mean ± SD; if not, they were shown as median (quartile). For categorical factors, results are shown as percentages. The chi-squared test or Fisher’s exact test was employed to analyze categorical data, while the Student’s t test or Mann–Whitney U test was utilized to assess disparities in baseline features of continuous variables among groups. Collinearity diagnostics were used to detect the presence of multicollinearity between independent variables. Binary logistic regression analysis for risk factors for post-thrombolysis END. To evaluate the overall discriminative power of the AIP and NHHR for post-thrombolysis END, a receiver operating characteristic (ROC) curve was created using a MedCalc 15.6.0 (MedCalc Software Acacialaan 22, B-8400 Ostend, Belgium) packet program. A two-tailed value of *p* < 0.05 was considered significant.

## Results

### Characteristics of clinical and demographic factors in AIS patients experiencing post-thrombolysis END compared to non-END

**Figure 2:**
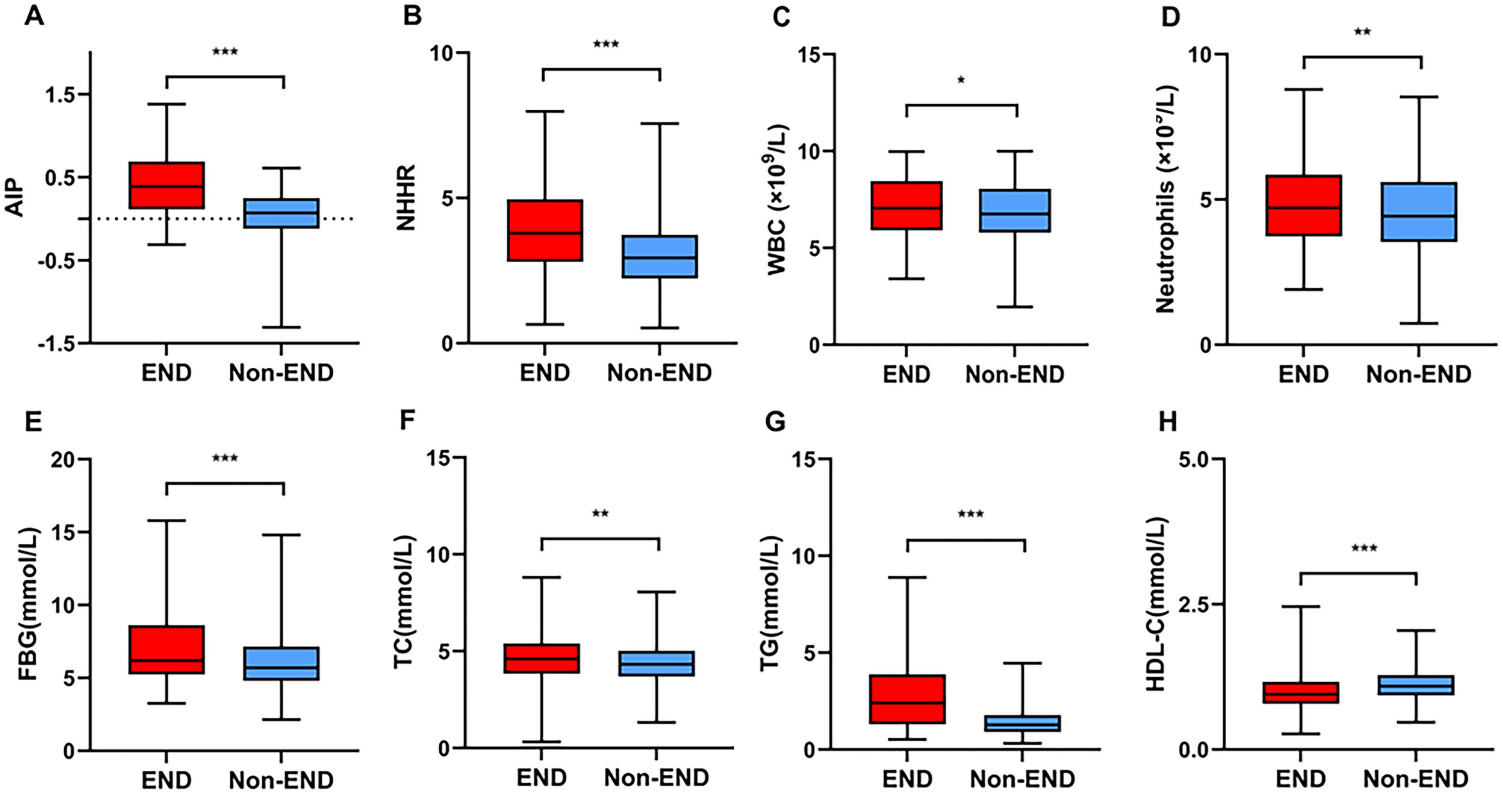
Comparisons of the AIP **(A)**, NHHR **(B)**, WBC **(C)**, neutrophils **(D)**, FBG **(E)**, TC **(F)**, TG **(G)**, and HDL-C **(H)** between the END and non-END groups. ^***^p < 0.001, ^**^p < 0.01, ^*^p < 0.05.

Table 1 provides the baseline information of all research participant. This study identified post-thrombolysis END in 198 patients (14.97%) and non-post-thrombolysis END in 1,125 patients (85.03%). In the post-thrombolysis END group, the NIHSS score after rt-PA for 24 h (*p* < 0.001), diabetes mellitus (*p* = 0.003), WBC (*p* = 0.014), neutrophils (*p* = 0.003), FBG (*p* < 0.001), TC (*p* = 0.001), TG (*p* < 0.001), AIP (*p* < 0.001),and NHHR (*p* < 0.001) were significantly greater than those in the post-thrombolysis non-END group, whereas HDL-C (*p* < 0.001) was significantly lower than those in the post-thrombolysis non-END group. The proportion of patients having symptomatic intracranial hemorrhage (sICH) in the post-thrombolysis END group was 26.77% (53/198). Furthermore, there was a significant difference in stroke subtype (*p* = 0.003) between the two groups. Figure 2 shows the AIP, NHHR, WBC, neutrophils, FBG, TC, TG, and HDL-C for the two groups.

**Table 1:** Characteristics of clinical and demographic factors in AIS patients experiencing post-thrombolysis END compared to non-END.

Variable	END (*n* = 198)	Non-END (*n* = 1,125)	*T*/*Z*	*p*
Demographic characteristics				
Age, years	67.30 ± 12.22	65.57 ± 12.55	−1.772	0.077
Male, *n* (%)	132 (66.67)	692 (61.51)	1.905	0.168
BMI, kg/m^2^	23.43 ± 4.60	22.97 ± 4.03	−1.179	0.239
Clinical assessment				
NIHSS, score at admission	6 (3–11)	6 (3–12)	−0.271	0.786
NIHSS, score after rt-PA 24 h	7 (3–13)	3 (1–8)	−6.623	<0.001
sICH, *n* (%)	53 (26.77)			
SBP, mmHg	150.28 ± 23.61	147.87 ± 20.47	−0.905	0.366
DBP, mmHg	86.21 ± 13.46	83.29 ± 12.91	−1.774	0.077
OTT, minute	157 (111–215)	145 (86–213.5)	−1.237	0.216
Lesion location, *n* (%)			1.803	0.401
Anterior circulation	148 (74.75)	889 (79.02)		
Posterior circulation	50 (25.25)	236 (20.98)		
Both	10 (5.05)	59 (5.24)		
Vascular risk factors, *n* (%)				
Hypertension	122 (61.62)	723 (64.27)	0.513	0.474
Diabetes mellitus	56 (28.28)	214 (19.02)	8.889	0.003
Atrial fibrillation	27 (13.64)	183 (16.27)	0.872	0.350
Coronary artery disease	36 (18.18)	249 (22.13)	1.555	0.212
Current smoking	89 (44.95)	449 (39.91)	1.771	0.183
Current drinking	45 (22.73)	244 (21.69)	0.106	0.744
Medication use history, *n* (%)				
Previous antiplatelet	24 (12.12)	161 (14.31)	0.671	0.413
Previous anticoagulation	15 (7.58)	83 (7.38)	0.010	0.922
Previous statin	13 (6.57)	84 (7.47)	0.201	0.654
Previous antihypertension	78 (39.39)	452 (40.18)	0.043	0.836
Previous hypoglycemic agents	27 (13.64)	136 (12.09)	0.373	0.541
Stroke subtype, *n* (%)			15.813	0.003
LAA	84 (42.42)	357 (31.73)		
SAO	75 (37.88)	531 (47.20)		
CE	24 (12.12)	188 (16.71)		
SOE	5 (2.53)	11 (0.98)		
SUE	10 (5.05)	38 (3.38)		
Laboratory data				
WBC (×10^9^/L)	7.45 (6.19–9.09)	7.05 (5.88–8.54)	−2.454	0.014
Hb (×10^9^/L)	135 (124–149)	134 (123–147)	−0.978	0.328
Platelets (×10^12^/L)	198.5 (169.5–242.3)	201 (164–239)	−0.123	0.902
Neutrophils (×10^9^/L)	4.99 (3.81–6.59)	4.57 (3.6–5.91)	−2.932	0.003
Lymphocytes (×10^9^/L)	1.64 ± 0.71	1.75 ± 0.86	1.776	0.076
Monocytes (×10^9^/L)	0.45 ± 0.23	0.44 ± 0.42	−0.137	0.891
FBG (mmol/L)	6.50 (5.25–9.92)	5.71 (4.81–7.25)	−4.801	<0.001
TC (mmol/L)	4.61 (3.85–5.39)	4.32 (3.69–5.02)	−3.318	0.001
TG (mmol/L)	2.46 (1.33–4.15)	1.27 (0.91–1.78)	−11.086	<0.001
HDL-C (mmol/L)	1.00 ± 0.29	1.13 ± 0.32	5.197	<0.001
LDL-C (mmol/L)	2.76 ± 1.01	2.74 ± 0.88	−0.246	0.805
AIP	0.39 (0.11–0.69)	0.07 (−0.12–0.25)	−11.115	<0.001
NHHR	2.94 (2.23–3.73)	3.83 (2.79–4.96)	−7.798	<0.001

BMI, body mass index; sICH, symptomatic intracranial hemorrhage; SBP, systolic blood pressure; DBP, diastolic blood pressure; NIHSS, National Institutes of Health Stroke Scale; OTT, onset to treatment time; LAA, large-artery atherosclerosis; SAO, small-artery occlusion; CE, cardioembolism; SOE, stroke of other determined etiology; SUE, stroke of undetermined etiology; WBC, white blood cells; TC, total cholesterol; TG, triglycerides; FBG, fasting blood glucose; HDL-C, high-density lipoprotein cholesterol; LDL-C, low-density lipoprotein cholesterol; AIP, atherogenic index of plasma; NHHR, non-high-density lipoprotein cholesterol to high-density lipoprotein cholesterol ratio.

### Analysis of logistic regression for risk factors associated with post-thrombolysis END

**Figure 3:**
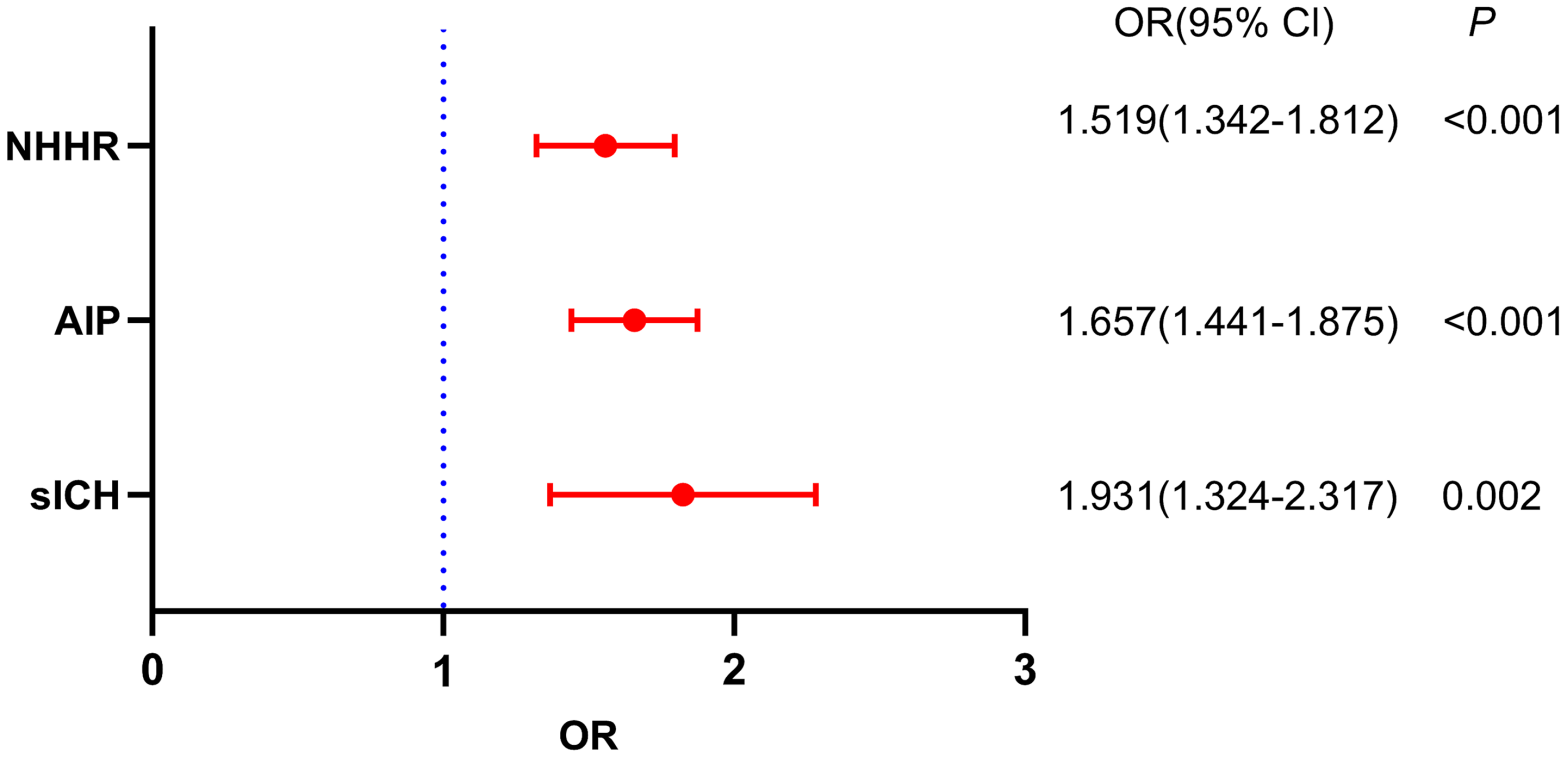
Binary logistic analysis of independent risk factors related to post-thrombolysis END.

Table 2 shows the outcomes of the post-thrombolysis END crude models. The binary logistic regression model incorporated the statistically significant variables from Table 1 and determined independent risk factors for post-thrombolysis END. Multivariate analysis should incorporate sICH, age, and the NIHSS score, as these are significant determinants for post-thrombolysis END. There was no collinearity between the AIP and the NHHR (VIF = 1.3). However, TC (VIF = 35), TG (VIF = 43), and HDL-C (VIF = 29) were not included in the model because of collinearity with the AIP and NHHR. After controlling for age, initial NIHSS score, diabetes mellitus, stroke subtype, WBC, neutrophils and FBG, the AIP (OR, 1.657; 95% CI 1.432–1.875, *p* < 0.001), NHHR (OR, 1.519; 95% CI 1.342–1.811, *p* < 0.001) and sICH (OR, 1.931; 95% CI 1.324–2.317, *p* = 0.002) were found to be independent predictors for post-thrombolysis END (Figure 3). The AIP and NHHR were utilized as a classification variable based on tertiles. Following the adjustment for confounding variables, patients exhibiting elevated AIP levels (3th quartile vs. 1st quartile; OR, 1.925; 95% CI, 1.325–2.437, *p* = 0.005) and NHHR levels (3rd quartile vs. 1st quartile; OR, 1.853; 95% CI, 1.274–2.564, *p* = 0.009) demonstrated a heightened risk of post-thrombolysis END (Table 3).

**Table 2:** Analysis of logistic regression for risk factors associated with post-thrombolysis END.

Variable	OR (95% CI)	*p*	Adjusted OR (95% CI)	*p*
Age	1.275 (1.097–1.375)	0.077	1.099 (0.993–1.026)	0.264
Initial NIHSS score	1.198 (1.077–1.318)	0.816	1.009 (0.974–1.025)	0.948
sICH	2.976 (2.234–3.645)	<0.001	1.931 (1.324–2.317)	0.002
Diabetes mellitus	1.615 (1.274–2.327)	0.023	1.257 (1.031–1.923)	0.915
LAA	Reference		Reference	
SAO	0.897 (0.523–1.950)	0.007	0.930 (0.613–0.981)	0.784
CE	0.540 (0.248–1.174)	0.005	0.867 (0.495–1.220)	0.120
SOE	0.495 (0.210–1.168)	0.010	0.560 (0.039–0.929)	0.108
SUE	0.511 (0.383–1.763)	0.556	0.641 (0.425–0.879)	0.653
WBC	1.106 (1.045–1.171)	0.001	1.070 (0.991–1.155)	0.082
Neutrophils	1.031 (1.006–1.057)	0.017	1.018 (1.009–1.325)	0.301
FBG	1.166 (1.113–1.222)	<0.001	1.137 (1.076–1.202)	0.053
TC	1.199 (1.085–1.313)	0.002		
TG	2.984 (2.501–3.561)	<0.001		
HDL-C	0.455 (0.280–0.898)	0.003		
AIP	1.995 (1.705–2.229)	<0.001	1.657 (1.423–1.875)	<0.001
NHHR	1.748 (1.537–1.989)	<0.001	1.519 (1.342–1.811)	<0.001

**Table 3:** Association between AIP, NHHR, and post-thrombolysis END.

Variable	OR (95% CI)	*p*	Adjusted OR (95% CI)^a^	*p*
AIP ternary classification				
T1	Reference		Reference	
T2	1.695 (1.382–2.304)	<0.001	1.403 (1.013–2.015)	0.032
T3	2.473 (1.931–3.526)	<0.001	1.925 (1.325–2.437)	0.005
NHHR ternary classification				
T1	Reference		Reference	
T2	1.632 (1.398–1.998)	<0.001	1.401 (1.112–1.984)	0.041
T3	2.225 (1.874–2.945)	<0.001	1.853 (1.274–2.564)	0.009

AIP: T1 ≤ −0.07; −0.07 < T2 ≤ 0.29; T3 > 0.29. NHHR: T1 ≤ 2.37; 2.37 < T2 ≤ 3.81; T3 > 3.81. ^a^Model: adjusted for age, initial NIHSS score, diabetes mellitus, stroke subtype, WBC, neutrophils, FBG, and TC.

### Subgroup and sensitivity analyses

Stratified analyses were conducted to determine if the relationship between AIP or NHHR and post-thrombolysis END was not influenced by some of the subgroups (Supplementary Figure 1). Our subgroup analyses revealed that the positive correlation was not significantly altered by stratification variables including age, sex, hypertension, diabetes mellitus, atrial fibrillation, coronary artery disease, smoking status, and alcohol use. AIP and NHHR were significantly associated with post-thrombolysis END in all subgroups, indicating that our study conclusion is robust and applicable to a wide range of populations. However, future external validation is warranted.

### ROC curve analysis was conducted to assess the overall capacity to distinguish post-thrombolysis END

**Figure 4:**
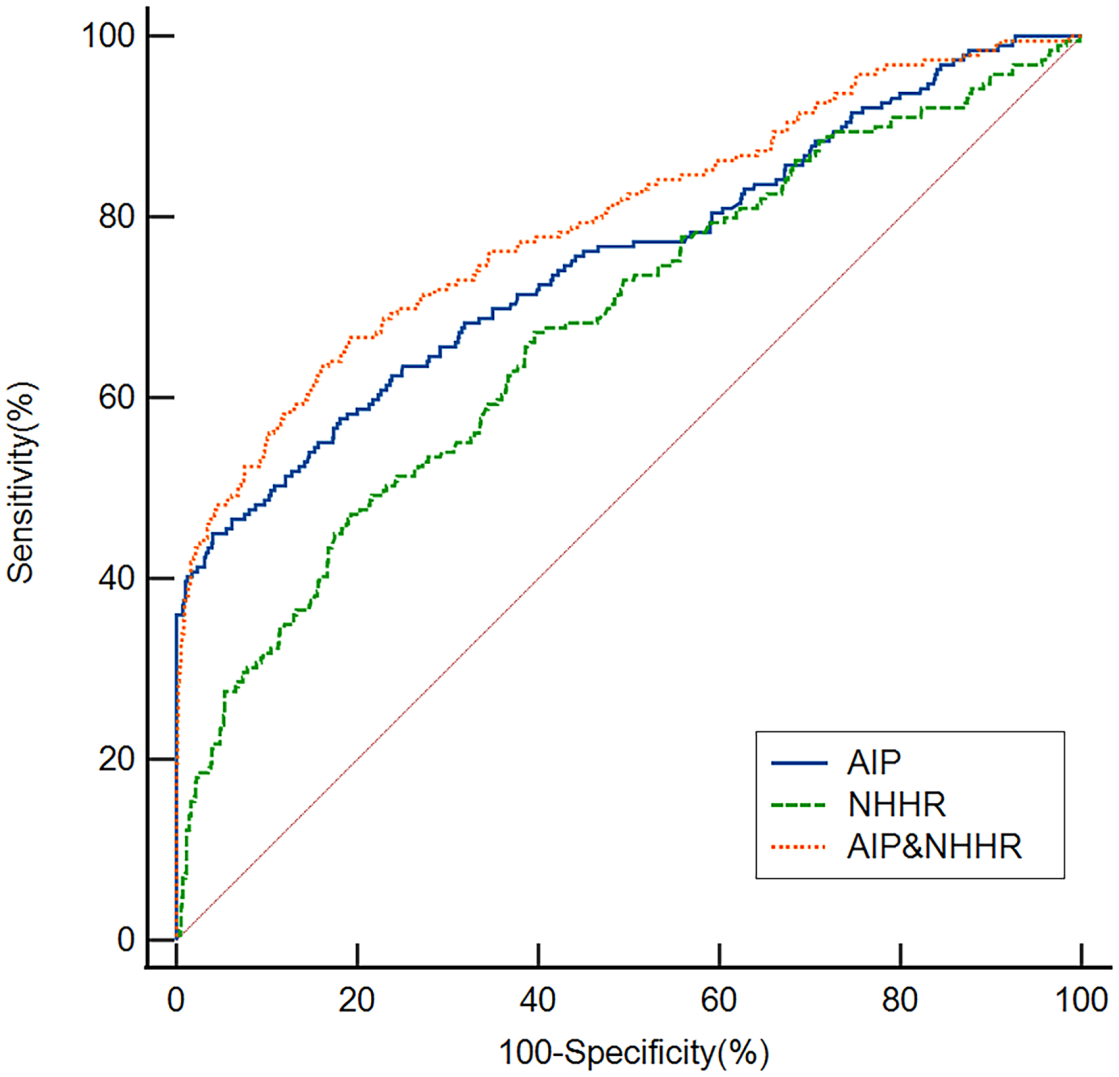
Based on ROC analysis, the AIP, NHHR and AIP and NHHR exhibited respectable post-thrombolysis END discriminating power with AUC values of 0.754, 0.678, and 0.795, respectively.

As seen in Figure 4, ROC curves were used to assess the AIP and NHHR’s overall discriminatory capacity in differentiating post-thrombolysis END. The AIP’s area under the curve (AUC) for determining post-thrombolysis END was 0.754 (95% CI, 0.729–0.778, *p* < 0.001), and the cut-off value was 0.46, with a sensitivity of 45.26% and a specificity of 95.99%. For the NHHR, the AUC was 0.678 (95% CI, 0.651–0.705, *p* < 0.001), and the cut-off value was 3.946, with a sensitivity of 47.09% and a specificity of 80.72%. Additionally, we conducted ROC curve analysis to assess the discriminatory power of the AIP and NHHR combination in distinguishing between the END group and the non-END group. The AUC for the combination of the NHHR and AIP was 0.795 (95% CI: 0.771–0.818, *p* < 0.001), and the cut-off value was 0.18, with a sensitivity of 66.67% and a specificity of 80.72%.

## Discussion

The pathophysiology of END following a stroke is not completely understood. According to research, it might be intimately linked to the development of thrombosis, the shedding of atherosclerotic plaque, inadequate collateral circulation, oxidative stress, and inflammation (35). There is no particular treatment for END in clinical practice once it happens. Hence, the capacity of doctors to recognize stroke patients at high risk of END at an early stage is crucial.

This research represents the inaugural investigation into the relationship between AIP and post-thrombolysis END in AIS patients. 198 individuals (14.97%) in this study developed post-thrombolysis END, and the percentage matched those from other investigations (5, 6, 36). This investigation yielded some novel discoveries. Initially, we found that the AIP and NHHR in AIS patients with END were greater than those in AIS patients with non-END. Additionally, the binary logistic regression model indicated that the AIP and NHHR were independent factors for post-thrombolysis END. Lastly, according to ROC analysis, the AIP and NHHR showed decent post-thrombolysis END discriminating power. Together, these findings provide evidence that a higher AIP and NHHR are associated with post-thrombolysis END. The findings indicate that a higher AIP and NHHR correlate with post-thrombolysis END.

The AIP, a unique comprehensive lipid index that may accurately reflect the ratio of atherogenic to anti-atherogenic lipid particles, has lately drawn the attention of researchers (37). A meta-analysis indicated that elevated AIP serves as an independent prognostic factor in individuals with coronary artery disease (38). A previous study demonstrated that AIP serves as an independent factor for no-reflow in patients with acute myocardial infarction undergoing primary percutaneous coronary intervention (39). A higher cumulative AIP was significantly associated with an increased risk of major adverse cardiac events, stroke and myocardial infarction independent of traditional cardiovascular risk factors (40). The Kailuan Study, which included 97,959 participants, demonstrated a significant association between high baseline and long-term AIP levels and an increased risk of ischemic stroke (41). An elevation in baseline AIP levels was significantly correlated with risk for ischemic stroke in middle-aged and elderly populations (1). Furthermore, an elevation in baseline AIP levels was significantly correlated with risk for ischemic stroke in middle-aged and elderly populations (29). Our study found that the AIP was significantly higher in the post-thrombolysis END group compared to the non-END group. A binary logistic regression model demonstrated that the AIP was an independent predictor of post-thrombolysis END. Moreover, when the AIP was employed as a categorical variable, our analysis, after controlling for confounding variables, indicated that an elevated AIP correlated with an increased likelihood of developing post-thrombolysis END. The findings suggest that elevated AIP during the acute phase of ischemic stroke may serve as a risk factor for post-thrombolysis END. Furthermore, the AIP demonstrated commendable post-thrombolysis END discriminative capability with AUC values of 0.754. These data may suggest that the AIP serves as a biomarker for post-thrombolysis END.

We suggested that this phenomenon could be explained by a number of possible mechanisms. First of all, the AIP reflects atherogenic dyslipidemia characterized by high TG, low HDL cholesterol, and the sdLDL, a subcomponent of LDL with pro-inflammatory and pro-atherogenic characteristics (11). TG rich lipoproteins, such as very low-density lipoprotein or chylomicron, are sufficiently small to penetrate the arterial intima (42, 43). These changes induce sustained low-grade inflammation and foam cell accumulation, culminating in atherosclerotic plaque destabilization and rupture (44). Additionally, sdLDL can promote the overproduction of reactive nitrogen and reactive oxygen species, thereby exacerbating endothelial cell damage (45). AIP indicates insulin resistance, which is closely associated with glucose metabolic dysfunction (46). Brain edema, symptomatic bleeding, and the progression of ischemia could result from these pathophysiological alterations (47). Finally, a variety of earlier studies have found strong correlations between AIP and a number of associated conditions, such as coronary heart disease, diabetes mellitus, hypertension, and current smoking, all of which have been identified as significant risk factors for END (48–51). These results highlight how AIP may mediate the existence of comorbidities and hence accelerate the progression of stroke.

The NHHR is a developing comprehensive metric of atherosclerotic lipids (52). Prior research has demonstrated that when assessing the risk of non-alcoholic fatty liver disease (53) and chronic kidney disease (54), the NHHR performs better in terms of predictive and diagnostic capabilities than conventional blood lipid levels. Moreover, numerous recent studies have highlighted NHHR’s predictive worth and its strong prognostic value in people with cardiovascular disease (23–26). A prior study indicated that NHHR is associated with an increased prevalence of stroke and may become a new predictor of stroke (27). There are currently no studies that precisely examine how NHHR predicts the risk of post-thrombolysis END or how it is directly related to post-thrombolysis END. In this study, the NHHR was significantly higher in the post-thrombolysis END group compared to the non-END group. A binary logistic regression model demonstrated that the NHHR was an independent predictor of post-thrombolysis END. When the NHHR was employed as a categorical variable, it after controlling for confounding variables, indicated that an elevated NHHR correlated with an increased likelihood of developing post-thrombolysis END. Furthermore, the NHHR demonstrated commendable post-thrombolysis END discriminative capability with AUC values of 0.678. The findings suggest that elevated NHHR during the acute phase of ischemic stroke may serve as a risk factor for post-thrombolysis END. This connection can be explained pathophysiologically by the part lipoproteins play in atherosclerosis (55). Several studies have shown that non-HDL cholesterol, which comprises all atherogenic lipoproteins carrying apolipoprotein B, is more strongly linked to cardiovascular risk than LDL cholesterol alone (56, 57). On the other hand, because HDL cholesterol makes it easier for the body to eliminate cholesterol, it protects against atherosclerosis (58, 59). As a result, a higher NHHR indicates a greater proportion of atherogenic particles than protective ones, which may raise the risk of atherosclerotic events like post-thrombolysis END.

Our study’s sICH percentage in the END group was 26.77% (53/198), which is comparable to a previous study (33). Additionally, all AIS patients receiving thrombolysis had an overall sICH rate of 4.01% (53/1,323), which is in accordance with previous research (6, 34, 60). Similar to the previous study (6, 61), sICH was found to be an independent risk factor for post-thrombolysis END in this investigation after controlling for all other variables. Numerous studies exist on the early prediction of END. These factors include inflammation (neutrophil-to-Lymphocyte ratio, hypersensitive C-reactive protein, interleukin-6), protease (matrix metalloproteinase-9, alkaline phosphatase, lipoprotein-associated phospholipase A), coagulation (P-selectin and C-type lectin-like receptor 2, D-Dimer), metabolism (blood glucose, glycated albumin, blood Lipid, cystatin C, whole blood purine concentration, trimethylamine N-Oxide), oxidative stress and excitatory neurotoxicity (F2-Isoprostanes, microRNA-107, serum total bilirubin) (35). Additionally, the triglyceride-glucose (TyG) index and TG/HDL-C ratio are emerging as promising candidates for post-thrombolysis END (6). Other significant indicators for END include age, the NIHSS score, hypertension and hypotension, infarct location and large artery occlusion (28, 62). Owing to the vast heterogeneity in stroke progression, a single biomarker is unlikely to accurately predict the risk of progression. Combining multiple biomarkers is therefore necessary to enhance the capacity to estimate END. AIP and NHHR are easy to obtain markers in clinical practice and can be widely used. Moreover, no study to date has investigated the association between AIP/NHHR and END following intravenous thrombolysis in Asian AIS patients. This study indicates that the AIP and NHHR may serve as prognostic factors for predicting post-thrombolysis END. Our findings expand the understanding of the function of the AIP and NHHR in cerebrovascular disease and provide fresh perspectives on therapeutic approaches. However, our research found no significant correlation between age, the NIHSS score, and post-thrombolysis END. We think that the differences between various studies could be due to differences in the ethnicity of the research populations, sample sizes, medication status, and the severity of the condition. Furthermore, the AIP and NHHR demonstrated statistically significant AUC values, the practical clinical usefulness (especially given the moderate sensitivity) should be further explored. In our study, the AIP AUC = 0.754, but sensitivity = 45.26% limits its standalone screening value. Combined AIP + NHHR model yields AUC = 0.795. So, the combination of these two indicators may be clinical risk scores or decision tools in the future.

This study has the following limitations: (1) This study was cross-sectional and involved only Chinese patients receiving intravenous thrombolysis, indicating the presence of potential inherent biases. Moreover, there was a difference in sample size between END (*n* = 198) and non-END (*n* = 1,125) patients. Consequently, our findings necessitate verification in non-Chinese populations, and future research should involve larger-scale longitudinal cohort studies; (2) only the baseline AIP and NHHR levels were tested. There were no dynamic AIP and NHHR changes accessible during the hospital period; (3) because the data on the participants’ medicine (lipid-lowering, antihypertensive, and hypoglycemic medications) and lifestyle (physical activity, smoking status, and alcohol use) were self-reported, they were susceptible to recall bias; (4) this paper does not show the details of the infarction, including location and volume; (5) although numerous potential confounders were taken into account, the results could still have been impacted by unmeasured or residual confounders; and (6) there was no specific time of blood sample collection after intravenous thrombolysis. Future research needs to consider the specific time of blood sample collection after intravenous thrombolysis.

## Conclusion

In conclusion, this study indicates that the AIP and NHHR may serve as prognostic factors for predicting post-thrombolysis END. The combination of these two indicators may be more beneficial in predicting post-thrombolysis END. Further research is necessary to validate these findings and elucidate the pathophysiology of post-thrombolysis END.

## Data Availability

The raw data supporting the conclusions of this article will be made available by the authors, without undue reservation.

## Supplementary material

SUPPLEMENTARY FIGURE 1 Subgroup analyses of AIP, NHHR and post-thrombolysis END.
