# Supplementary figures and images for: Association between the atherogenic index of plasma and the non-high-density lipoprotein cholesterol to high-density lipoprotein cholesterol ratio with early neurological deterioration after thrombolysis
Source: Front Neurol. 2025 Aug 28;16:1619727. doi: 10.3389/fneur.2025.1619727 (PMC12422903; doi:10.3389/fneur.2025.1619727)

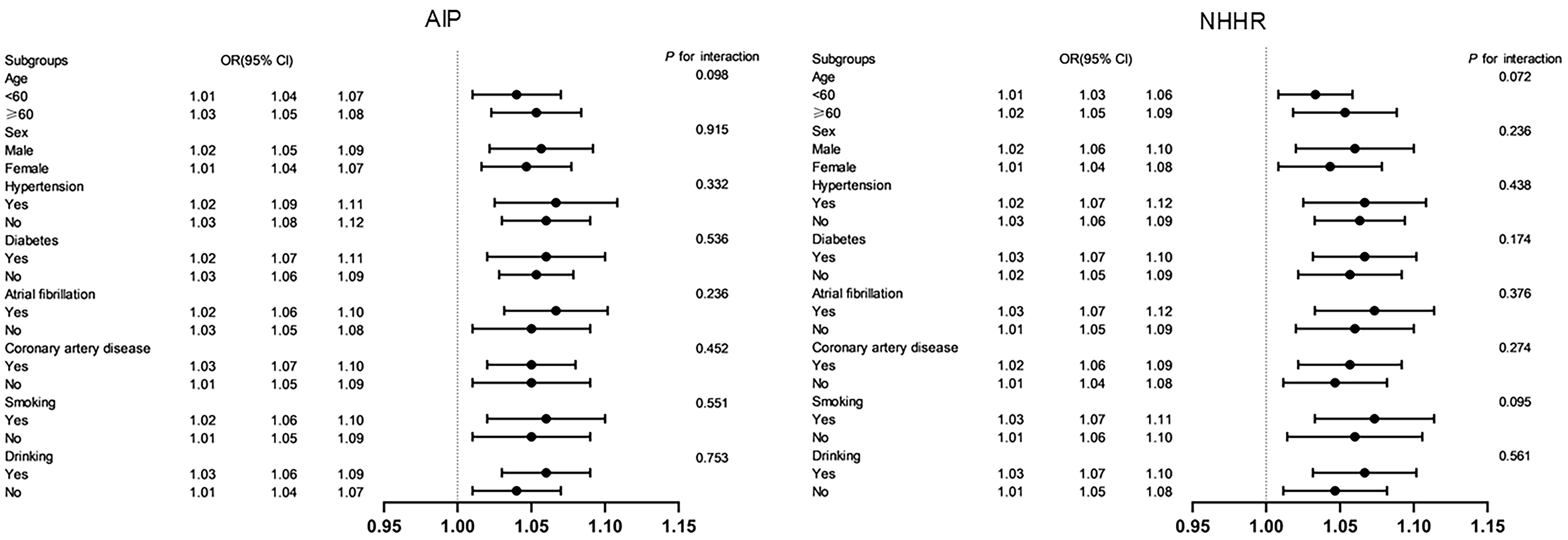

Supplement: SUPPLEMENTARY FIGURE 1 — Subgroup analyses of AIP, NHHR and post-thrombolysis END. [file Image_1.tif]
